# Dexrazoxane Shows No Protective Effect in the Acute Phase of Reperfusion during Myocardial Infarction in Pigs

**DOI:** 10.1371/journal.pone.0168541

**Published:** 2016-12-21

**Authors:** Pranitha Kamat, Stijn Vandenberghe, Stephan Christen, Anjan K. Bongoni, Bernhard Meier, Robert Rieben, Ahmed A. Khattab

**Affiliations:** 1 Department of Clinical Research, University of Bern, Bern, Switzerland; 2 ARTORG Center, University of Bern, Bern, Switzerland; 3 Department of Infectious Diseases, University of Bern, Bern, Switzerland; 4 Department of Cardiology, Bern University Hospital, Bern, Switzerland; Klinikum Region Hannover GmbH, GERMANY

## Abstract

Calcium and iron overload participate in the mechanisms of ischemia/reperfusion (I/R) injury during myocardial infarction (MI). Calcium overload induces cardiomyocyte death by hypercontraction, while iron catalyses generation of reactive oxygen species (ROS). We therefore hypothesized that dexrazoxane, an intracellular metal chelator, would attenuate I/R injury. MI was induced in pigs by occlusion of the left anterior descending artery for 1 hour followed by 2 hours reperfusion. Thirty minutes before reperfusion either 5 mg/ml dexrazoxane (n = 5) or saline (n = 5) was infused intravenously. Myocardial necrosis as percentage of the area at ischemic risk was found to be similar in both groups (77.2 ± 18% for dexrazoxane and 76.4 ± 14% for saline group) as determined by triphenyl tetrazolium chloride staining of the ischemic myocardium. Also, serum levels of troponin-I were similar in both groups. A conductance catheter was used to measure left ventricular pressure and volume at all times. Markers for tissue damage due to ROS (HNE), endothelial cell activation (CD31) and inflammation (IgG, C3b/c, C5b9, MCP-1) were assessed on tissue and/or in serum. No significant differences were observed between the groups for the parameters analyzed. To conclude, in this clinically relevant model of early reperfusion after acute myocardial ischemia, dexrazoxane lacked attenuating effects on I/R injury as shown by the measured parameters.

## Introduction

Although mortality rates of acute myocardial infarction have considerably declined over the past decades [1,2,3], the risk of heart failure and death remains high [1]. Prompt recanalization of the infarct-related artery, preferably by percutaneous coronary intervention (PCI), to re-establish myocardial perfusion comprises the state-of-the-art treatment. However, paradoxically, reperfusion can induce injury and thereby contribute to the adverse outcome after myocardial infarction. It is therefore vital to aid the ischemic heart in confronting the consequences of reperfusion, protecting it from ischemia/reperfusion (I/R) injury.

The current understanding of the mechanisms of I/R injury is that it has a multifactorial etiology, with several synergistic pathways contributing towards tissue necrosis. Among these, the generation of reactive oxygen species (ROS) [4,5] and calcium overload appear to be important. Reperfusion reintroduces oxygen to the previously ischemic tissue which results in a sudden deleterious burst of ROS, including superoxide anion, hydrogen peroxide and hydroxyl radicals [6] [7]. Other sources of ROS generation include anaerobic mitochondrial respiration [8,9], activated neutrophils [10,11] and certain enzymes [12]. Detection of ROS during myocardial infarction in humans and attenuation of myocardial infarction in animal models by antioxidants, demonstrate the significance of ROS [13,14].

Among the different ROS generated, hydroxyl radical is a strong oxidant. It is not scavenged specifically by any of the available biological antioxidants, and leads to peroxidation of lipid membranes [15]. In biological systems it is formed in the presence of transition metals, in particular by iron which acts as catalyst [16]. Since iron propagates the effects of ROS, neutralizing it may be effective in reducing tissue damage during I/R [17] as suggested by reduction of myocardial infarct size by chelating the iron with desferioxamine [18].

Abrupt increase in intracellular calcium also occurs during reperfusion. This results in cardiac cell death due to hypercontracture and opening of the mitochondrial permeability transition pore [19,20]. Thus, reduction in free calcium could also attenuate injury following ischemia and reperfusion.

Dexrazoxane is a clinically approved drug and is used in the US and Europe to reduce anthracyclin-induced cardiotoxicity [21,22,23,24]. It is a bis-dioxopiperazine that is intracellularly hydrolyzed to ADR-925 [25,26] and was proven to reduce redox activity of iron [26,27] and calcium by chelation [28]. The free iron binding capacity of dexrazoxane has also been shown under hypoxic conditions [29]. We therefore hypothesized that Dexrazoxane would attenuate I/R injury and tested this hypothesis in a clinically relevant model of myocardial infarction in pigs. The pig model adopted in this study was designed to simulate myocardial I/R as it would occur in a patient. The study gives prime importance to the duration of ischemia, i.e. from the incidence of occlusion of a coronary artery, to the earliest point in time when the patient seeks medical attention. Furthermore, to make the study clinically relevant, a clinically approved dosage of dexrazoxane was used. The effects of dexrazoxane as an attenuator of acute myocardial I/R injury was tested until 2 hours of reperfusion, and was shown to have no protective effects.

## Materials and Methods

Care and use of animals in the present study were in compliance with the Guide for the Care and Use of Laboratory Animals (NIH publication no. 85–23, revised 1996) as well as Swiss National Guidelines. The local ethical committee for animal research (Amt für Landwirtschaft und Natur des Kantons Bern) approved the conduction of this study. Source of the pigs was a local farm in Bern, Switzerland, which is registered with the responsible authorities as breeder of laboratory animals.

### Closed-chest porcine model of myocardial infarction

Ten medium-sized German Landrace pigs (30 ± 2 kg) were pre-medicated with ketamine (20 mg/kg), midazolam (0.5 mg/kg) and atropine (0.05 mg/kg), intubated, and mechanically ventilated with a Draeger respirator (O_2_/air 1:2, isoflurane 1.5–2.5 vol.%). Continuous infusions of pancuronium bromide (0.4 mg/kg/h, i.v.) and fentanyl (10 μg/kg/h, i.v.) were given throughout the procedure. ECG, central venous pressure and mean arterial pressure were monitored throughout the experiment with a Hewlett-Packard CMS patient monitor. A single bolus of unfractionated heparin (5000 IU) was administered intravenously before starting the procedure. Heparin (2500 IU) was administered intravenously every two hours thereafter.

The pigs were placed in recumbent position, followed by surgical exposure of left and right external carotid arteries and right jugular vein. Vascular access sheaths were introduced in each vessel prior to catheterization. The left anterior descending (LAD) artery was occluded just distally of the first large diagonal branch with a semi-compliant conventional PCI balloon catheter (Maverick, Boston Scientific, Natick, MA, USA; balloon length 20 mm, diameter 3.0 mm). The balloon was inflated to completely occlude the vessel (Encore 26 inflation device, Boston Scientific; used pressure 4–6 atm). Localization of the balloon and state of inflation was controlled angiographically on a regular basis using a BV PULSERA digital mobile C-arm fluoroscopy system (Philips Medical System).

Thirty minutes after the onset of ischemia 80 ml of 5 mg/ml Dexrazoxane (Cardioxane^®^, Novartis, Switzerland, hereafter Dex), (n = 5) or 80 ml saline (n = 5) were infused intravenously at the rate of 8 ml/min. One hour after onset of ischemia, the balloon was deflated and removed, to allow for 2 hours of reperfusion. At the end of the reperfusion period, the balloon was re-introduced and re-inflated at the same place in the LAD and 60 ml Evan’s Blue (Sigma-Aldrich Chemical Company, Ltd. St. Louis, MO, USA; 2% wt/vol solution) was injected intravenously. The pigs were sacrificed with an intravenous 10 ml bolus of 20% potassium chloride just after Evan’s Blue injection and the heart was excised for further analysis.

All experimenters were blinded with regard to treatment assignment. Randomization of the animals into the two groups was done using a randomization code with a random number generator prior to the beginning of each experiment. The 80 ml samples were prepared according to the randomization output on the morning of each experiment by an independent laboratory technician. Sample size was determined in advance, estimated from previous experience and not by formal sample size calculations.

In the case of ventricular fibrillation during the experiment, a biphasic defibrillator (150 J) was used for cardioconversion.

### Myocardial area at risk and necrosis

The excised heart was cut perpendicular to the septum from the apex to the base into 3 mm slices until the mitral valve. For every slice the Evan’s Blue unstained parts within the left ventricle were separated, weighed and expressed as a percentage of the left ventricle. This gave the myocardial area at ischemic risk (AAR) as percentage of the left ventricle. The AAR from all slices were then treated with 1% triphenyl tetrazolium chloride (TTC, Sigma, pH 7.4) for 20 min at 37°C. Viable myocardium [viable ischemic tissue (VIT)] was stained bright red while the infarcted tissue [necrotic ischemic tissue (NIT)], remained unstained. High dynamic range images of the slices were taken after the TTC staining. The TTC unstained areas within the AAR were marked by ImageJ software and expressed as percentage of the AAR to obtain the myocardial necrosis/infarct size. Tissue samples were taken from the Evan’s Blue stained areas that were not at ischemic risk (ANR), NIT and VIT for immunohistochemical staining.

### Measurement of left ventricular function using pressure-volume loops

A conductance catheter (GX 5 system, Scisence Inc., London, Ontario) was placed in the left ventricle via the left carotid artery to measure left ventricular pressure and volume. Blood resistivity of each individual pig was measured at the beginning of the experiment for calibration. The catheter was positioned in the long axis so that left ventricular pressure-volume loops displayed stable rectangular shapes. LV pressure and volume, ECG, central venous pressure, and arterial pressure were all recorded and digitized at 200Hz with an acquisition device (model 416, iWorx, Dover, NH) at all times during the experiment. Analysis of heart function was done with the Labscribe2 software (iWorx Systems Inc., Dover, NH) at time points of baseline, 50 min of ischemia, 1 hour and 2 hours of reperfusion. For each time point, hemodynamic parameters were calculated per heart cycle and averaged over at least 20 sequential heartbeats. Also energetic parameters were calculated: stroke work as the energy transferred to the ejected blood, potential energy as the energy required for muscle contraction, and pressure-volume area as the sum and thus the total energy consumed by the left ventricle.

### Detection of myocardial necrosis, vascular damage and inflammation in blood serum

Systemic venous blood was collected directly into tubes containing serum clot activator (S-Monovette, Sarstedt, Nümbrecht, Germany) and kept at 4°C. Tubes were centrifuged at 3000 rpm for 10 min and the serum collected as supernatant stored at –80°C until used. Samples were taken at baseline after injection of heparin, 50 min of ischemia, 10 min, 1 hour and 2 hours of reperfusion. Samples were analyzed for markers of myocardial necrosis, vascular damage and inflammation by a multiplex assay with use of a Bio-Plex assay system (Bio-Rad).

Commercially available antibodies specific for porcine cardiac Troponin (cTn)-I (mAb, HyTest), CD31 (mAb, R&D), MCP-1 (pAb, PeproTech), C3b/c (pAb, Dako) and C5b-9 (mAb, Diatec) were used as capture antibodies. Each antibody was coupled to different fluorescently labeled polystyrene COOH Luminex-beads by using Bio-Plex amine coupling kit (Bio-Rad). The amount of captured analytes was then measured by using biotinylated detection antibodies, followed by streptavidin-PE, or sheep anti-FITC PE for C3b/c detection. Calibration curves from recombinant protein standards were prepared with threefold dilution steps in antibody diluent containing 0.5% polyvinyl alcohol and 0.8% polyvinylpyrrolidone. Standards were measured, and blank values were subtracted from all readings. A mixture containing 2500 coupled beads per antibody (50 μl/well) was incubated with the standards, sample and blank in a final volume of 50 μl/well. The mix was incubated for 60 min at room temperature. Beads were washed three times, and incubated together with a cocktail of detection antibodies: anti cTn- I-biotin (pAb, HyTest), anti CD31-biotin (mAb, AbD Serotec), anti MCP-1-biotin (pAb, PeproTech), anti C3b/c-FITC (pAb, Dako) and anti C6-biotin (mAb, Quidel) in a final volume 25 μl/well. After 30 min incubation with detection antibody, the beads were washed and a mixture (50 μl/well) of streptavidin-PE (Qiagen) and Sheep anti- FITC-PE (pAb, Antibodies-online) were added. The detection antibodies, streptavidin-PE and anti FITC-PE were all added at a final concentration of 1 μg/ml. After 10 min incubation and washing, measurement and data analysis were performed with the Bio-Plex system and Bio-Plex Manager software.

### Tissue markers for complement activation and damage due to ROS

Immunofluorescence staining was done by the free float technique in tissue samples obtained from three regions of the excised heart, i.e. ANR, VIT and NIT. Samples from a normal pig’s heart that did not undergo any procedure were also included. Samples were fixed in 2% formaldehyde for 24 hours followed by overnight incubation in 18% sucrose at 4°C. Samples were then embedded in Shandon M1 embedding matrix and stored at -20°C until staining.

Tissues were sectioned at 30 μm thickness and permeabilized with Triton-X100 for 15 min at room temperature. Sections were then incubated with the directly labeled primary antibody, goat anti porcine IgG FITC (Southern Biotech 6050–02) for 90 min at RT. The unlabeled primary antibodies rabbit anti-C3b/c (Dako A0062), rabbit anti-C5b9 (Calbiochem 204903) and rabbit anti-HNE (Alpha diagnostic HNE 11-S) were incubated overnight at 4°C. The secondary antibody used was sheep anti-rabbit Cy3 (Sigma C2306).

Sections from myocardial tissue of a normal pig were used as negative controls and for normalizing staining intensity between experiments. Representative images were taken with a confocal microscope from the stained sections. Histograms were obtained with ImageJ software and normalized to obtain the area under the curve (AUC) values. Sections were finally compared based on the AUC, avoiding inter assay errors caused by differences in staining intensity. The experimenter was blinded with respect to treatment groups during sectioning, staining and analysis by ImageJ.

### Statistical analysis

For analyzing the time dependent changes in heart function and for markers in the serum, each time point was expressed as a percentage of its baseline value and plotted as a line graph for every pig. Area under the curve (AUC) values were obtained from the line graphs and used to compare the groups with the two-tailed Mann-Whitney test. One-Way ANOVA and Bonferroni post-test were used to compare immunofluorescence staining between tissue samples. Two-tailed unpaired t test was used to compare the groups at a given time point or for a single tissue type.

## Results

During the experimental procedure, two out of five pigs treated with Dex suffered from ventricular fibrillation and required defibrillation. This occurred in one pig during ischemia and in the other pig once during ischemia and once during early reperfusion. In the saline group, three out of five pigs had to be defibrillated. Two times during ischemia for one pig, one time during ischemia and four times during reperfusion for the second pig and one time during ischemia for the last pig.

### Myocardial area at risk and necrosis

**Fig 1:**
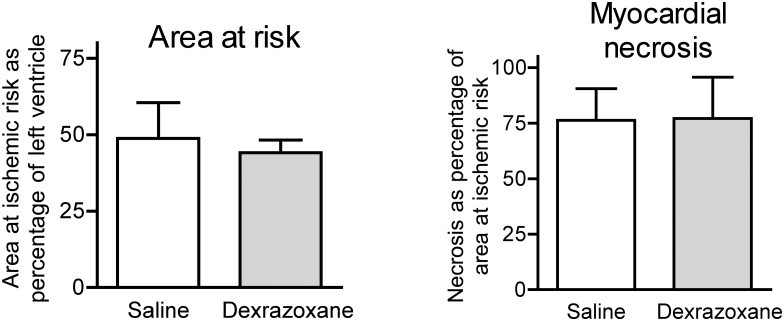
Myocardial area at risk and necrosis. Myocardial area at ischemic risk was compared by expressing the area at ischemic risk as percentage of the left ventricle. Myocardial necrosis was determined by TTC staining and expressed as percentage of the area at ischemic risk.

The area at ischemic risk (AAR) was expressed as a percentage of the left ventricle and was comparable in both groups (Fig 1). This suggests that the extent of ischemic injury induced was similar between the groups. The mean ± standard deviation values were 44 ± 4% for the Dex group and 48.8 ± 11% for the saline group. The myocardial necrosis calculated as percentage of the AAR, was 77.2 ± 18% for the Dex group and 76.4 ± 14% for the saline group (Fig 1). Treatment with Dex therefore had no effect on myocardial necrosis as detected by macro staining of the myocardium.

### Left ventricular function

Cardiac function was assessed during the course of the experiment by a conductance catheter placed in the left ventricle. Absolute values of heart rate, minimum volume, end diastolic volume, maximum pressure, potential energy, pressure volume area, stroke volume and stroke work are shown in Table 1 as mean ± standard deviation. For two animals, the loops recorded were not stable and were therefore excluded from the analysis. The stroke volume was measured to be lower than physiological values. This is because calibration with a reference volume of flow was not performed. Mean value for stroke volume in the saline group is higher during ischemia than during baseline. However, differences are not statistically significant due to high standard deviations. Overall, the maximum pressure seemed to be higher in the saline group, but also for this parameter the differences between groups were not statistically significant.

**Table 1:** Left ventricular function.

Parameter	Dexrazoxane (n = 4)				Saline (n = 4)				p-value^a^
	Baseline	Ischemia	1h Reperf.	2h Reperf.	Baseline	Ischemia	1h Reperf.	2h Reperf.	
Heart rate (bpm)	127.7±14.5	99.3±18.7	134.4±24.3	120.3±13.6	115.1±6.1	100.4±16.8	120.7±18.3	113.5±13.6	0.68
Min volume (ml)	45.8±10.4	45.8±10.0	40.9±11.4	44.9±11.2	40.1±6.2	44.9±8.9	34.8±10.7	37.8±13.6	0.88
End diast vol (ml)	59.4±11.6	62.7±11.1	58.6±9.1	61.7±9.1	54.2±7.7	59.8±7.9	51.7±7.2	51.1±11.8	0.20
Max press (mmHg)	106.3±8.1	81.7±19.8	73.6±14.9	81.4±11.4	96.2±14.5	88.5±21.2	73.7±4.0	91.6±23.3	0.20
Pot energ (watt.sec)	251.4±63.2	187.4±45.3	153.1±26.7	186.5±15.8	157.9±135.1	161.7±86.3	90.8±29.9	130.0±20.3	0.20
Press vol (watt.sec)	403.6±114.1	314.4±54.8	275.1± 47.6	320.4±71.1	293.0±193.8	266.5±115.2	202.3±80.5	230.2±29.0	0.20
Stroke vol (ml)	11.5±4.5	11.7±5.4	14.5±7.3	11.4±3.3	12.7 ±2.6	17.2±6.6	16.9±6.3	16.1±5.6	0.20
Stroke work (watt.sec)	1049.1±341.8	919.8±302.7	875.9±409.0	967.0±483.3	998.1±475.8	749.4±189.3	792.6±360.8	755.5±178.7	0.34

Values are mean ± standard deviation.

^a^ p-value determined by two-tailed Mann-Whitney U test

### Detection of myocardial necrosis, endothelial dysfunction and inflammation in blood serum

**Fig 2:**
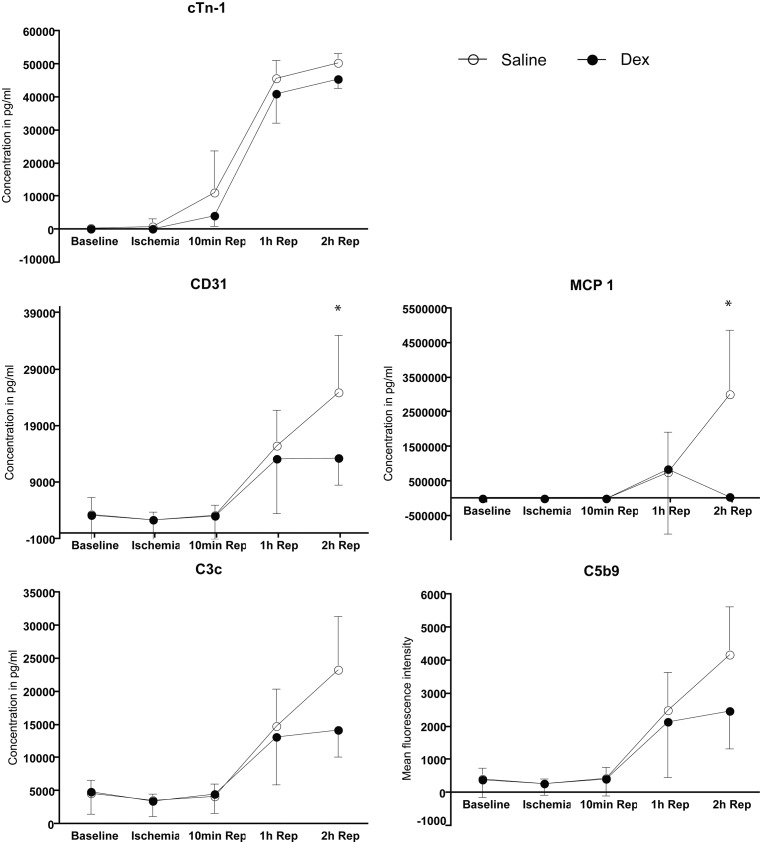
Detection of myocardial necrosis, endothelial dysfunction and inflammation in blood serum. Blood serum samples obtained at baseline, 50min after ischemia, 10min, 1hour and 2hours after reperfusion were analyzed. for myocardial necrosis by measuring cardiac troponin-I. Endothelial dysfunction was analyzed by measuring CD31 protein levels and inflammation was analyzed by measuring MCP-1, C3b/c and C5b9 levels.

Serum samples were analyzed for cardiac troponin-I (cTn-I) as a marker for myocardial necrosis (Fig 2). At baseline, cTn-1 was measured to be 436±483 pg/ml (saline group) and 323±410 pg/ml (Dex group), respectively. During ischemia it was 1114±2126 pg/ml and 251±274 pg/ml in the saline and Dex groups. respectively. At 10 min, 1 hour and 2 hours of reperfusion, the values in the saline group were 11379±12546 pg/ml, 45740±5358 pg/ml and 50568±2652 pg/ml, respectively. The values in the Dex group for these time points were 4189±3253 pg/ml, 41069±8801 pg/ml and 45548±2882 pg/ml, respectively. Mann-Whitney U-test suggested no difference in overall levels of cTn-I between the groups.

The platelet endothelial cell adhesion molecule-I (CD31) was measured as a marker for endothelial dysfunction (Fig 2). At baseline it was measured to be 3390±2959 pg/ml in the saline group and 3304±4878 pg/ml in the Dex group. During ischemia it was measured to be 2422±1421 pg/ml and 2429±3580 pg/ml in saline and Dex groups, respectively. At 10 min, 1 hour and 2 hours after the onset of reperfusion, CD31 in the saline group gradually increased with 3294±1791 pg/ml, 15519±6372 pg/ml and 24926±10124 pg/ml, respectively. In the Dex group it was increased from 10 min (3146±4172 pg/ml) to 1 hour reperfusion (13271±9720 pg/ml). At 2 hours reperfusion the CD31 levels were maintained at 13340±4751 pg/ml in the Dex group. The Mann-Whitney U-test showed no overall difference between the groups. However, at 2 hours after reperfusion the CD31 level was significantly higher in the saline group when compared to the Dex group as tested by two-tailed unpaired t-test (p = 0.0077).

To assess the extent of inflammation, monocyte chemotactic protein-I (MCP-1) was measured in saline and Dex group, and was found to be 0.3±0.2 pg/ml and 0.5±0.5 pg/ml at baseline, respectively (Fig 2). During ischemia, it was 0.2±0.1 pg/ml in the saline group and 0.3±0.2 pg/ml in the Dex group. MCP-1 levels were maintained at 10 min of reperfusion with 0.4±0.2 pg/ml in the saline group and 0.4±0.4 pg/ml in the Dex group. The levels gradually increased at 1 hour and 2 hours after reperfusion in the saline group with 76±117 pg/ml and 301±187 pg/ml, respectively. In the Dex group, the MCP-1 levels increased at 1 hour (85±187 pg/ml) and then decreased (6±5 pg/ml). Over time from baseline to reperfusion, no difference was observed in the MCP-1 levels between the two groups as tested by Mann-Whitney U-test. The increase in MCP-1 at 2 hours reperfusion in saline group was significantly higher than that in the Dex group as tested by two-tailed unpaired t-test (p = 0.0492).

The soluble form of complement factor C3b/c was measured as another marker for inflammation (Fig 2). At baseline, the values in the saline group were 4688±2039 pg/ml and 4889±3359 pg/ml the Dex group. During ischemia it was measured to be 3675±866 pg/ml (saline group) and 3528±2319 pg/ml (Dex group). During reperfusion the levels of C3b/c increased from 4215±1830 pg/ml (10min), to 14844±5608 pg/ml (1 hour) and 23357±8028 pg/ml (2 hours) in the saline group and from 4576±2949 pg/ml (10min), to 13153±7152 pg/ml (1hour) and 14292±4137 pg/ml (2 hours) in the Dex group. The Mann-Whitney U-test suggested no differences between the groups.

Soluble membrane attack complex of complement, sC5b9, was also measured as a marker for inflammation and was 417±331 pg/ml (saline group) and 402±544 pg/ml (Dex group) at baseline (Fig 2). During ischemia it became 287±129 pg/ml (saline group) and 285±343 (Dex group). The levels of sC5b9 were similar between the two groups at 10 min of reperfusion 458±321 pg/ml (saline group) and 417±493 pg/ml (Dex group) and at 1 hour after reperfusion 2501±1147 pg/ml (saline group) and 2144±1681 pg/ml (Dex group). At 2 hours of reperfusion sC5b9 was 4184±1443 pg/ml (saline group) and 2490±1146 pg/ml (Dex group). The overall serum levels for C5b9 were similar in both groups, as tested by the Mann-Whitney U- test.

### Tissue staining for antibody, complement deposition and damage due to ROS

**Fig 3:**
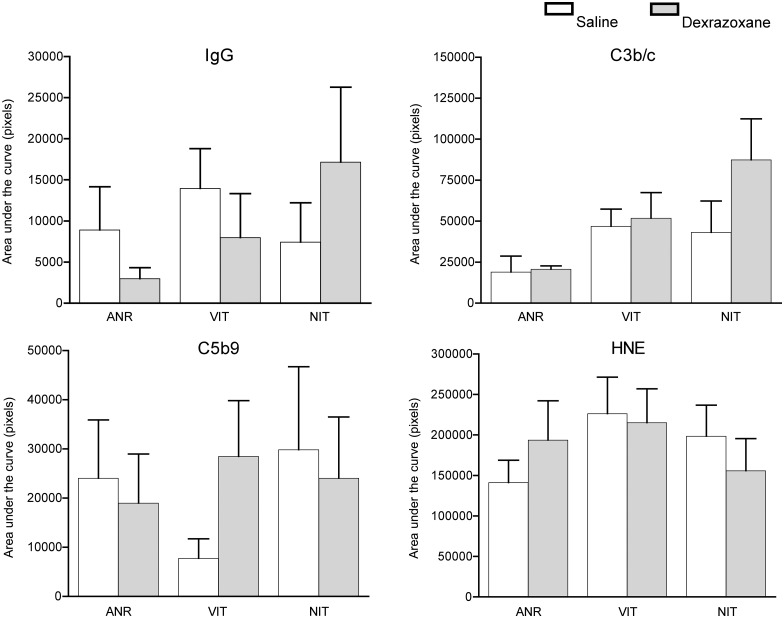
Tissue staining for antibody, complement deposition and damage due to ROS. Tissue samples were obtained from different sections of the myocardium including area not at ischemic risk (ANR), viable ischemic tissue (VIT) and necrotic ischemic tissue (NIT). The samples were then stained for IgG antibody deposition, C3b/c and C5b9 complement deposition. Damage to the myocardial tissue due to reactive oxygen species was analyzed by staining for 4-hydroxynonenal.

**Fig 4:**
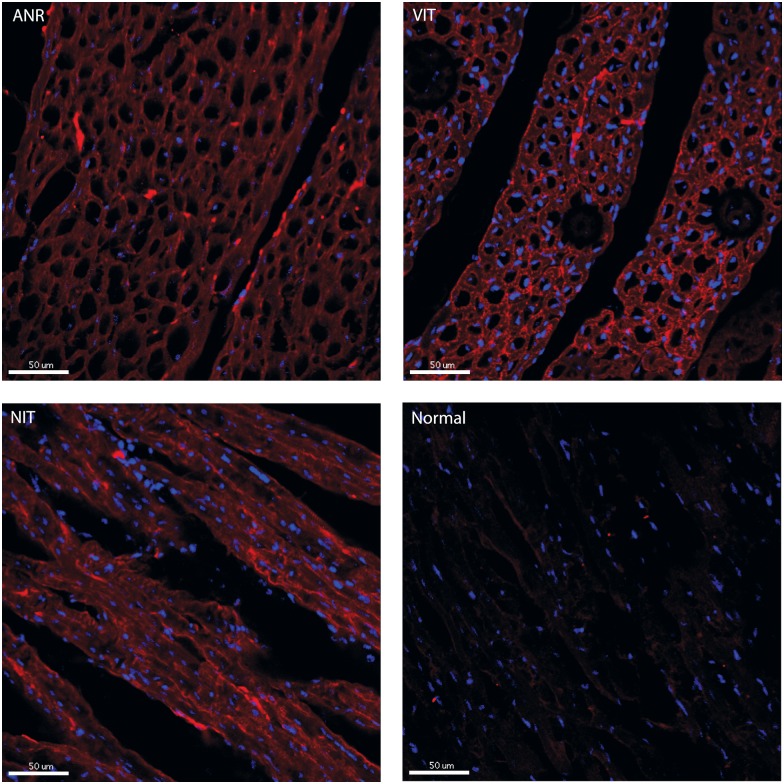
Representative pictures for HNE staining. Representative images of 4-hydroxynonenal staining on myocardial tissue from area not at ischemic risk (ANR), viable ischemic tissue (VIT) and necrotic ischemic tissue (NIT) are shown. As control, a normal myocardial tissue that was not subjected to ischemia reperfusion injury was used. Red = HNE, blue = DAPI staining of nuclei. Scale bars = 50μm.

To assess the extent of inflammation on tissue, complement activation as analyzed by deposition of IgG, C3b/c and C5b-9. Tissues taken from different regions of area not at ischemic risk (ANR), viable ischemic tissue (VIT) and necrotic ischemic tissue (NIT) were compared between the two groups. No significant differences were found between the groups in the different tissue types. Similar results were obtained for staining for 4 hydroxynonenal (HNE), which was used as a marker for tissue damage due to ROS. Data are shown in Fig 3 and representative pictures for HNE staining are shown in Fig 4.

## Discussion

The aim of this study was to evaluate the effectiveness of Dex in a clinically relevant animal model of acute myocardial infarction. The findings of the study indicate no favorable effects of Dex as revealed by both functional and biological assays within the first 2 hours of reperfusion after a 1 hour ischemic period.

Clinical relevance and adequacy of experimental model: In clinical practice a patient with acute myocardial infarction encountering first medical contact before 30 minutes of chest pain onset (i.e. vessel closure) is rare, which is also reflected in the definition of acute myocardial infarction (chest pain at rest for longer than 20 minutes). In our study we therefore administered Dex 30 min after ischemia. The dosage we used was also adopted from clinical practice, being the clinically approved dosage for prevention of anthracyclin-induced cardiomyopathy, which was derived from dose-finding studies. Although the toxic dose for Dex is higher (1000 mg/m^2^) [22], this does not mean that adverse and serious adverse events would not occur with lower doses, which is again of great importance if this regimen would be adopted in clinical practice. An in vivo model was essential as the activity of ROS is known to differ between in vivo and ex vivo models [30]. Pigs are known to have lower collateral flow than humans, nonetheless for cardiology experiments pig models are preferred over smaller animals for their anatomical similarities to human heart. Lastly, the current model of closed chest myocardial infarction in pigs has been the basis of several studies in our laboratory, wherein the positive attenuating effects of dextran sulfate, APT070 and tyrosine sulfate on myocardial I/R injury could be confirmed [31,32,33].

This study hypothesized that Dex as a chelator of iron and calcium would attenuate ROS mediated tissue necrosis during myocardial infarction. The results obtained from this study suggest the hypothesis to be untrue. In our model, Dex had no effect on reducing myocardial necrosis and enhancing cardiac function as determined by the infarct size, cTn-I levels and left ventricular function. In current clinical practice, cTn-I measurements are conducted until 72 hours of reperfusion to identify myocardial necrosis. The short reperfusion duration of 2 hours is thus a limitation in the current study. The deposition of HNE as a marker for damage due to ROS showed no significant differences between the Dex treated and untreated animals. ROS mediated endothelial dysfunction was measured by levels of CD31 in the serum [34,35]. Although no differences were seen overall, at 2 hours of reperfusion the Dex group showed significantly lower levels of CD31 than the control group. ROS induces MCP-1 expression in cardiomyocytes and endothelial cells [36,37,38], even in the absence of infarction [39], to recruit leukocytes [40]. Chen et al. have previously shown reduced expression of MCP-1 in the absence of ROS and iron [41]. In our study, no significant differences were seen between the groups in the overall levels of MCP-1. However, a significantly lower level of MCP-1 in the Dex group was observed at 2 hours of reperfusion when compared to the saline group. Other markers of inflammation including deposition of IgG, C3b/c and C5b9 in the tissue and in circulation showed no significant differences between groups. Altogether, assessment of the above parameters revealed no effect of Dex in ROS induced I/R injury.

The ineffectiveness of Dex in our study contradicts studies conducted in the past and can be explained by the experimental model and protocols used. Ramu et al., used the Langendorff system to perfuse isolated rat hearts with Krebs-Henseleit buffer during the course of experimental I/R injury, and showed attenuating effects of Dex [42]. This model lacks the important local and systemic effects of I/R, caused mainly by the inflammatory and coagulatory cascades in vivo. The concept as well as our data is further supported by a study that compared in vivo and ex vivo rat models to test the effects of Dex during myocardial infarction [43]. This study showed effective reduction in infarct size of hearts treated with 150 mg/kg Dex ex vivo but not in vivo. The effectiveness of Dex in vivo was achieved only when the dose was increased to 450 mg/kg. This could explain the results from our study wherein a low dosage of 14 mg/kg was chosen, to ensure clinical applicability. Before its use in the clinics, Dex has proven to be a cardioprotectant when administered intraperitoneally 30 min before induction of myocardial damage [44]. The hydrolyzed metabolite of Dex that carries the iron chelating action was detected, within 2 min of i.v. administration in rats, with highest levels detected in the heart [45]. The i.v. injection of Dex in our study therefore ensures rapid availability of the metabolite in the myocardium.

A cascade of deleterious reactions including inflammation and generation of reactive oxygen species is initiated at the onset of ischemia and is continued during early reperfusion. These cascade reactions in the early phase of reperfusion contribute largely to myocardial necrosis (summarized in [46,47]) and intervening clinically at this phase could provide cardioprotection. On the other hand, progressive reperfusion contributes towards cardiac tissue repair also known as cardiac remodeling. However, the process of cardiac remodeling by itself carries deleterious effects and is the leading cause for heart failure after myocardial infarction. Effectively regulating the process of tissue repair could substantially reduce the deleterious effects of remodeling [47]. The current study aimed to attenuate I/R injury by pharmacologically intervening with Dex during the early reperfusion phase. The study shows no attenuating effects of Dex until 2 hours after reperfusion, but lowered levels of CD31 and MCP-1 were observed at the end of 2 hours reperfusion. It could therefore be that Dex is an effective treatment during cardiac remodeling rather than a cardio-protectant during the initial reperfusion phase. Furthermore, since ROS plays a critical role during cardiac remodeling it could serve as a potential target for Dex. Further studies are required to test this hypothesis with longer reperfusion periods and check for delayed effectiveness of Dex treatment.

In previous studies, Dex was shown to be more effective when administered immediately prior to or along with the anthracyclin treatment [22]. We therefore conducted experiments (results not shown) where 2 mg/kg Dex was injected directly into the ischemic area (via the tip of the balloon catheter) 10 min before the onset of reperfusion. The first pig died during infusion because of intractable ventricular fibrillation and therefore the infusion rate of Dex was made as low as possible in the next two pigs. Inspite of the low infusion rate the two pigs were hemodynamically unstable during infusion and therefore further experiments with this protocol had to be terminated. The histological examination for these 2 pigs showed infarct areas no different to the control group.

To conclude, in this clinically relevant model of myocardial infarction with 1 hour ischemia and 2 hours of reperfusion, the tested parameters have shown dexrazoxane to be ineffective in protecting the myocardium against I/R injury.
